# Risk of head and traumatic brain injuries associated with antidepressant use among community-dwelling persons with Alzheimer’s disease: a nationwide matched cohort study

**DOI:** 10.1186/s13195-017-0285-3

**Published:** 2017-08-01

**Authors:** Heidi Taipale, Marjaana Koponen, Antti Tanskanen, Piia Lavikainen, Reijo Sund, Jari Tiihonen, Sirpa Hartikainen, Anna-Maija Tolppanen

**Affiliations:** 1 Kuopio Research Centre of Geriatric Care, University of Eastern Finland, PO Box 1627, 70211 Kuopio, Finland; 2 School of Pharmacy, University of Eastern Finland, PO Box 1627, 70211 Kuopio, Finland; 3 Department of Forensic Psychiatry, Niuvanniemi Hospital, University of Eastern Finland, Kuopio, Finland; 4 Research Centre for Comparative Effectiveness and Patient Safety (RECEPS), University of Eastern Finland, PO Box 1627, 70211 Kuopio, Finland; 5 Department of Clinical Neuroscience, Karolinska Institutet, Tomtebodavägen 18A, 5th floor, 171 77 Stockholm, Sweden; 6 Impact Assessment Unit, National Institute for Health and Welfare, PO Box 30, 00271 Helsinki, Finland; 7 Centre for Research Methods, Department of Social Research, University of Helsinki, P.O. Box 54, 00014 Helsinki, Finland; 8 Department of Psychiatry, Kuopio University Hospital, PO Box 100, 70029 Kuopio, Finland

**Keywords:** Antidepressant, Head trauma, Traumatic brain injury, Older person, Alzheimer’s disease

## Abstract

**Background:**

Antidepressant use has been associated with an increased risk of falling, but no studies have been conducted on whether antidepressant use is associated with an increased risk of head injuries which often result from falling among older persons. The objective of this study was to investigate the risk of head and brain injuries associated with antidepressant use among community-dwelling persons with Alzheimer’s disease.

**Methods:**

A matched cohort study was conducted by comparing new antidepressant users (*n* = 10,910) with two matched nonusers (*n* = 21,820) in the MEDALZ study cohort. The MEDALZ cohort includes all community-dwelling persons newly diagnosed with Alzheimer’s disease between 2005 and 2011 in Finland. Incident antidepressant users were identified based on register-based dispensing data from the Prescription register with a 1-year washout period for antidepressant use. Nonusers were matched with users based on age, gender, and time since Alzheimer’s disease diagnosis. The outcome events were defined as any head injuries and traumatic brain injuries based on diagnoses in Hospital Discharge and Causes of Death registers. Propensity score adjusted Cox proportional hazard models were utilized. Sensitivity analyses with case-crossover design were conducted. All registers are linkable with unique personal identification numbers assigned for each resident.

**Results:**

Antidepressant use was associated with an increased risk of head injuries (age-adjusted event rate per 100 person-years 2.98 (95% confidence interval (CI) 2.49–3.06) during use and 2.43 (95% CI 2.06–2.35) during nonuse, adjusted hazard ratio (HR) 1.35, 95% CI 1.20–1.52) and traumatic brain injuries (age-adjusted event rate per 100 person-years 1.33 (95% CI 1.13–1.53) during use and 1.10 (95% CI 1.00–1.20) during nonuse, adjusted HR 1.26, 95% CI 1.06–1.50). The risk was highest during the first 30 days of use (HR 1.71, 95% CI 1.10–2.66 for head injuries; HR 2.06, 95% CI 1.12–3.82 for traumatic brain injuries) and remained at an elevated level for head injuries for over 2 years of use. In case-crossover analyses, antidepressant use was consistently associated with a higher risk of head injuries.

**Conclusions:**

Antidepressant use was associated with an increased risk of the most severe outcomes, head and brain injuries, in persons with Alzheimer’s disease. Antidepressant use should be carefully considered and the association confirmed in future studies.

**Electronic supplementary material:**

The online version of this article (doi:10.1186/s13195-017-0285-3) contains supplementary material, which is available to authorized users.

## Background

Alzheimer’s disease (AD), the most common form of dementia, is a major public health challenge due to population aging [1]. Age is the most important risk factor for AD. As AD leads to dependency on other people it is associated with significant health care and societal costs. For these reasons, optimal care of this vulnerable patient group is a key challenge for the future.

Antidepressant use is frequent among older persons and especially among persons with AD or other dementia [2–4]. In a Finnish AD cohort, prevalence of antidepressant use was 3.5-times more frequent among persons with AD than among persons without AD [4]. In addition, the incidence of antidepressant use peaks after the AD diagnosis, suggesting that antidepressants are frequently initiated for various symptoms among persons with AD [5]. Antipsychotics have been traditionally used for treatment of behavioral and psychological symptoms of dementia (BPSD), but their use has been associated with an increased risk of cerebrovascular adverse events and mortality [6]. Consequently, recent studies report decreasing use of antipsychotics together with increasing use of antidepressants [2, 7]. Due to more frequent use of antidepressants and changes associated with both aging and the AD disease process, investigating adverse drug events (ADE) associated with antidepressant use is crucially important in this population.

Among older persons, antidepressant use has been frequently associated with an increased risk of falls [8, 9], injurious falls like fractures [9], and hip fractures [10]. In addition, the risk for head and brain injuries increases with aging [11]. Among older persons, falls are the main causal factor for traumatic brain injuries (TBIs), whereas younger persons experience TBIs as a result of motor vehicle, sports, and other accidents [12]. Older persons (aged ≥65 years) are about two-times as likely to experience a TBI compared with younger persons [13]. Thus, fall-related head and brain injuries are a significant health problem among older persons. However, we found no studies investigating the risk of head or brain injuries associated with antidepressant use. One previous study assessed risk factors for TBIs during falls among older persons, but medication use was assessed only 4 h before the fall and the number of users was small [14].

The objective of our study was to investigate whether antidepressant use is associated with risk of head and brain injuries among community-dwelling persons with AD. We also studied the risk in terms of duration of use and compared the risk between selective serotonin reuptake inhibitors (SSRIs) and other antidepressants.

## Methods

### Cohort

The MEDALZ (Medication use and Alzheimer’s disease) cohort consists of all 70,718 community-dwelling persons diagnosed with AD between 2005 and 2011 in Finland. These persons were identified from the Special Reimbursement register [3, 15]. Current care guidelines in Finland recommend that all persons with clinically verified Alzheimer’s disease should be prescribed anti-dementia drugs if there is no contraindication for use [16]. The diagnostic process was conducted according to a predefined protocol which includes computed tomography or magnetic resonance imaging (MRI) scan according to the NINCDS-ADRDA [17] and DSM-IV criteria. A certificate of the fulfillment of the diagnostic criteria must be confirmed by a geriatrician or neurologist and sent for evaluation to the Social Insurance Institution of Finland which grants special reimbursement if the criteria are fulfilled.

### Registers

Data for the cohort have been collected from several nationwide registers including the Prescription register (years 1995–2012), the Special Reimbursement register (1972–2012), the Hospital Discharge register (1972–2012), and socioeconomic data since 1970 and causes of death 2005–2012 from Statistics Finland. All registers are linkable with unique personal identification numbers assigned for each resident. The Prescription register includes information on purchases of reimbursed drugs classified according to the Anatomical Therapeutic Chemical (ATC) classification system [18]. The purchased amount is recorded in the register as Defined Daily Dose (DDD), which is the assumed average maintenance dose per day for a drug used for its main indication in adults. Drug use data are restricted to community-dwelling persons since drugs used during stays in hospitals and public nursing homes are not recorded in the register. The Special Reimbursement register includes records of persons entitled for higher reimbursement of drugs due to chronic diseases. The Hospital Discharge register includes inpatient stays in hospitals with corresponding discharge diagnoses.

### Exposure

Antidepressants were defined according to ATC class N06A. SSRIs were defined as N06AB (fluoxetine, citalopram, paroxetine, sertraline, fluvoxamine, and escitalopram on the market in Finland). Other antidepressants (N06A excluding SSRIs N06AB) were grouped together. When drug use started and ended, i.e., drug use periods, were modeled with a previously utilized method, PRE2DUP [19]. The modeling is based on sliding averages of daily dose (in DDDs) and modeling separately each ATC code for each person according to purchase regularity taking into account hospitalizations, stockpiling of drugs, and changing dose. After modeling all antidepressant drugs separately, overlapping drug use periods of any antidepressant use were combined to retrieve continuous duration of any antidepressant use. For drug class analyses, SSRI-use periods were formed by combining drug use periods of SSRI drug substances together, and similarly for other antidepressants.

### Outcome

Diagnoses of head injuries and TBIs were collected from the Hospital Discharge register and Causes of Death register data. Thus, our study outcome represents head injuries and TBIs both treated in hospital and as a direct or underlying cause of death. Based on ICD-10 codes, head trauma was defined as S0* (injuries to the head), and TBIs were defined as S06 (intracranial injury). For each person, we considered only the first recorded outcome event (with the most specific diagnosis recorded in that period). Diagnoses of head and brain injuries before the AD diagnosis (with corresponding ICD-8 and -9 codes) were used for exclusion of persons with previous injury, and new diagnoses after AD were considered as the outcome of interest.

### Study setting

**Fig. 1:**
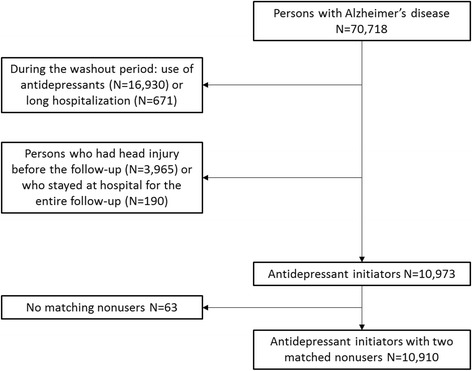
Flow chart of exclusions for this study

Exclusion criteria for this study were prevalent antidepressant use and long hospitalization during a 1-year washout period before AD diagnosis (Fig. 1). Persons using antidepressants, or who were hospitalized for >50% of the washout period or were hospitalized/institutionalized for >90 days at the end of the washout period, were excluded from all analyses. Exclusions due to long-term hospitalizations during the washout period were conducted because drug use during hospitalizations is not recorded in the Prescription register. Persons with previous head injury since 1972 until the AD diagnosis were excluded from all analyses. Furthermore, persons hospitalized/institutionalized for the entire follow-up period were excluded as the follow-up time for them never started.

After these exclusions, we constructed a matched cohort by selecting two nonusers as comparison persons for each person who started antidepressant use. The same exclusion criteria were applied for nonusers. At the start date of antidepressant use, two nonusers were matched for each user based on age (±2 years), gender, and time since AD diagnosis (variation allowed at maximum ±90 days), resulting in 10,910 antidepressant users and 21,820 matched nonusers. The purpose of this matching was to ensure that nonusers are similar to users especially in terms of time since AD diagnosis as a proxy for severity of illness. The same persons are included in both outcome analyses (head injury and TBI) and persons with head injury in TBI analyses were censored at the point when they experience head injury other than TBI. For 63 users, two comparison persons could not be identified and they were excluded from further analyses. The follow-up started on the date of antidepressant initiation for users and the corresponding matching date for nonusers.

### Covariates

Covariates potentially having an impact on the risk of head or brain injury and antidepressant use were considered as described in Additional file 1. Traditional multivariable analyses were adjusted for cardiovascular disease, diabetes, asthma/chronic obstructive pulmonary disease (COPD), epilepsy, substance abuse, cancer, schizophrenia, bipolar disorder or depression, baseline use of antipsychotics, opioids, benzodiazepines and related drugs, history of stroke and hip fracture, and socioeconomic position. In order to simultaneously control for a wider range of covariates that may have impact on initiation of antidepressants, we derived a propensity score by logistic regression. Propensity score represents the probability of treatment (antidepressant use) given the measured confounders that are included in the propensity score [20]. Propensity scores help to deal with the selection bias in which a number of observed (confounding) covariates might be unbalanced between the groups to be compared. This propensity score, including comorbid conditions (both somatic and psychiatric), use of antipsychotics, benzodiazepines and related drugs, and other drugs (ever before the follow-up or at the beginning of follow-up), and history of previous fractures, was used for adjusting. The covariates are described in Additional file 1 and all covariates presented in Table 1 (except age and gender, which were matching variables) were included in the propensity score. Socioeconomic class included missing data (no records at Statistics Finland) for about 1% of the cohort and this was coded as one category (unknown). Other variables did not include missing data.

**Table 1:** Comparison of antidepressant users and nonusers in terms of baseline characteristics, and unadjusted and propensity score adjusted logistic regression for differences

	Antidepressant nonuser (*n* = 21,820)	Antidepressant user (*n* = 10,910)	Unadjusted OR (95% CI)	Propensity score adjusted OR (95% CI)
Female gender (matching criteria)	69.0% (15,064)	69.0% (7532)	1.00 (0.95–1.05)	0.94 (0.90–1.00)
Aged ≥80 years (matching criteria)	51.6% (11,261)	51.4% (5613)	0.99 (0.95–1.04)	0.94 (0.90–1.00)
Socioeconomic position				
High	34.7% (7578)	33.5% (3659)	Reference	Reference
Medium	57.8% (12,618)	59.2% (6463)	1.06 (1.01–1.12)	1.04 (0.98–1.09)
Low	6.3% (1380)	6.0% (655)	0.98 (0.90–1.09)	0.92 (0.83–1.01)
Unknown	1.1% (244)	1.2% (133)	1.13 (0.91–1.40)	1.06 (0.85–1.33)
Comorbidities				
Cardiovascular disease	50.6% (11,043)	50.7% (5533)	1.00 (0.96–1.05)	1.00 (0.95–1.05)
Chronic heart failure	28.1% (6124)	29.8% (3215)	1.09 (1.03–1.14)	1.00 (0.95–1.06)
Cardiac arrhythmia	6.6% (1441)	7.4% (807)	1.13 (1.03–1.24)	1.00 (0.91–1.10)
Hypertension	30.0% (6543)	31.6% (3445)	1.08 (1.03–1.13)	1.00 (0.95–1.05)
Epilepsy	2.1% (451)	1.8% (198)	0.87 (0.74–1.04)	0.99 (0.83–1.17)
Asthma/COPD	8.4% (1837)	8.5% (925)	1.01 (0.93–1.10)	1.00 (0.92–1.09)
Any chronic pulmonary disease	10.4% (2258)	11.1% (1209)	1.08 (1.00–1.16)	1.00 (0.93–1.08)
Pulmonary circulation disorders	0.1% (19)	0.1% (11)	1.16 (0.55–2.44)	1.01 (0.48–2.15)
Diabetes	14.1% (3066)	12.8% (1395)	0.90 (0.84–0.96)	0.99 (0.93–1.06)
Complicated diabetes	16.1% (3506)	14.9% (1625)	0.91 (0.86–0.98)	0.99 (0.93–1.06)
Schizophrenia	1.3% (282)	1.1% (121)	0.86 (0.69–1.06)	0.97 (0.78–1.21)
Bipolar disorder/depression	1.8% (388)	2.7% (291)	1.51 (1.30–1.77)	1.00 (0.85–1.17)
Psychosis	3.7% (806)	5.7% (624)	1.58 (1.42–1.76)	1.00 (0.90–1.12)
Metastatic cancer	0.2% (41)	0.2% (18)	0.88 (0.51–1.53)	0.99 (0.56–1.74)
Any tumor	12.2% (2666)	13.0% (1420)	1.08 (1.00–1.15)	1.00 (0.93–1.08)
Renal failure	1.3% (273)	1.0% (109)	0.80 (0.64–1.00)	0.97 (0.77–1.22)
Hemiplegia	0.5% (99)	0.5% (59)	1.19 (0.86–1.65)	1.01 (0.72–1.40)
Coagulopathy	0.6% (135)	0.7% (78)	1.16 (0.87–1.53)	1.01 (0.75–1.34)
Liver disease	1.1% (231)	1.0% (110)	0.95 (0.76–1.20)	0.99 (0.79–1.26)
Peripheral vascular disorder	4.4% (958)	5.0% (550)	1.16 (1.04–1.29)	1.00 (0.90–1.12)
Anemia	6.8% (1485)	6.4% (693)	0.93 (0.85–1.02)	0.99 (0.90–1.09)
Fluid and electrolyte disorders	2.9% (633)	3.4% (367)	1.17 (1.02–1.33)	1.00 (0.87–1.14)
Alcohol abuse	1.1% (239)	1.5% (162)	1.36 (1.11–1.66)	1.01 (0.81–1.23)
Substance abuse	1.6% (343)	2.0% (218)	1.28 (1.08–1.52)	1.00 (0.84–1.19)
Previous hospital-treated fracture	19.1% (4165)	21.3% (2328)	1.15 (1.09–1.22)	1.00 (0.95–1.07)
Hip fracture	5.3% (1153)	6.2% (673)	1.18 (1.07–1.30)	1.00 (0.91–1.11)
Stroke	8.8% (1928)	10.1% (1097)	1.15 (1.07–1.25)	1.01 (0.93–1.09)
Drug use				
Drug use ever before the start of the follow-up				
Opioids	20.3% (4419)	24.6% (2678)	1.28 (1.21–1.35)	1.00 (0.95–1.06)
Antipsychotics	10.0% (2175)	10.5% (1147)	1.06 (0.98–1.15)	0.99 (0.92–1.07)
BZDRs	34.8% (7583)	45.4% (4952)	1.56 (1.49–1.64)	1.01 (0.96–1.07)
Antidepressants	13.5% (2942)	21.2% (2315)	1.73 (1.63–1.84)	1.01 (0.94–1.08)
Bisphosphonates	13.4% (2915)	14.3% (1560)	1.08 (1.01–1.16)	1.00 (0.93–1.07)
Anti-parkinson drugs	3.3% (727)	3.8% (410)	1.13 (1.00–1.28)	1.00 (0.88–1.14)
Antiepileptics	7.5% (1636)	8.9% (975)	1.21 (1.11–1.32)	1.00 (0.92–1.09)
Analgesics	78.9% (17,215)	82.1% (8952)	1.22 (1.15–1.30)	1.01 (0.95–1.07)
Cardiovascular drugs	84.0% (18,328)	85.5% (9327)	1.12 (1.05–1.20)	1.00 (0.94–1.07)
Drug use at the start of the follow-up				
Opioids	3.9% (851)	7.4% (805)	1.96 (1.78–2.17)	1.00 (0.90–1.11)
Antipsychotics	13.2% (2875)	20.7% (2260)	1.72 (1.62–1.83)	1.00 (0.94–1.08)
BZDRs	16.9% (3696)	31.0% (3379)	2.20 (2.09–2.32)	1.00 (0.92–1.08)

*BZDR* benzodiazepines and related drugs, *CI* confidence interval, *COPD* chronic obstructive pulmonary disease, *OR* odds ratio

### Statistical analyses

Analyses were restricted to the first antidepressant use periods as the new user design avoids prevalent user bias (i.e., prevalent users are the selected group as they tolerate the drug). Thus, the follow-up for users ended on discontinuation of use if that happened before other reasons for the end of follow-up. In all analyses, the follow-up ended on the outcome of interest (head injury or TBI), on >90 days hospitalization/institutionalization period, death, or the end of study follow-up (31 December 2012). In TBI analyses, the follow-up was censored if the person experienced a head injury other than TBI.

The analyses were conducted with Cox proportional hazard models by taking into account the matched design (own strata were used for each matching group). The main analyses compared antidepressant users with nonusers. Time-varying exposure was modeled using categorical time-dependent variable with classes for ≤30 days, 31–180 days, 181–365 days, 366–733 days, and over 733 days of exposure. Drug class analyses classified antidepressant users to SSRI users and other antidepressant users and compared these with nonusers. In drug class analyses, 38 persons starting concurrently with both SSRI and other antidepressant use were excluded and the analyses were censored if a person started concomitant use or switched the drug class during the follow-up. The drug class analyses were also restricted to the first 2000 days of use due to sparsity of data with a longer follow-up time. We conducted intention-to-treat (ITT) analyses to assess the effect of informative censoring, i.e., drug use is discontinued due to adverse effects that would lead to the studied outcome. In these ITT analyses, antidepressant users were considered as users for 180 days regardless of possible discontinuation of use or hospitalizations (the follow-up ended on outcome, death, and the end of study follow-up after which data were no longer available). The follow-up was restricted to the first 180 days as the initial intention for treatment was assumed to hold for only a certain time period. In as-treated analyses, comparison of users with nonusers was restricted similarly to the first 180 days of follow-up. All analyses were conducted unadjusted, adjusted for selected covariates, and adjusted for propensity score. The covariate balance across treatment groups before and after propensity score adjustment was assessed with logistic regression.

In addition, the dose-response relationship between antidepressant use and head injuries and TBIs was conducted by categorizing the mean dose in DDDs used in antidepressant monotherapy into <1 and ≥1 DDDs per day. Dose categories were compared to nonusers and by comparing higher dose with lower dose.

Sensitivity analyses with a case-crossover design were used with the aim to controlling for unmeasured confounding. Head and brain injury cases without exclusion of prevalent users were included in these within-individual analyses in which each person served as their own control. Persons with a previous head injury before AD diagnosis were excluded, and included persons needed to have at least 120 days of follow-up before the event. The case period was defined as 1–14 days before the outcome event (head injury or TBI), and three control periods were applied before the outcome (31–45, 60–74, and 120–134 days before the event). Multiple control periods were used to test the robustness of the results. Conditional logistic regression analyses were utilized to compare prevalence of antidepressant use between the case and control periods. The analyses were adjusted for time-dependent use of benzodiazepines and related drugs, antipsychotics, and opioids.

Statistical analyses were performed using SAS statistical software, version 9.3 (SAS Institute, Inc., Cary, NC, USA). Data were retrieved from the registers by the register maintainers and de-identified register data were submitted to the research team. Participants were not contacted in any way. According to Finnish legislation, no ethics committee approval is required in these circumstances.

## Results

In this study, 10,910 antidepressant users and 21,820 matched nonusers were included; the majority of these were women in both groups (69.0%). The mean age of antidepressant users was 79.5 (standard deviation (SD) 6.8) years and 79.6 (SD 6.7) years for nonusers. Table 1 shows the comparison between users and nonusers in terms of baseline characteristics. Antidepressant users were more likely to use other psychotropic drugs (antipsychotics and benzodiazepines and related drugs) and opioids and to have history of hospital-treated bipolar disorder or depression. None of the factors were associated with antidepressant use after adjusting for propensity score.

During the follow-up (median 249 days, interquartile range (IQR) 77–642 days, for antidepressant users and 656 days, IQR 316–1155, for nonusers), 1373 head injuries were recorded and 677 (49%) of them were TBIs. Age-adjusted incidence rate for head injuries per 100 person-years was 2.98 (95% confidence interval (CI) 2.49–3.06) during antidepressant use and 2.43 (95% CI 2.06–2.35) during nonuse (incidence rate difference 0.55, 95% CI 0.52–0.58). Age-adjusted incidence rate for TBIs per 100 person-years was 1.33 (95% CI 1.13–1.53) during antidepressant use and 1.10 (95% CI 1.00–1.20) during nonuse (incidence rate difference 0.23, 95% CI 0.21–0.25).

Antidepressant use was associated with an increased risk of head injury (propensity score adjusted hazard ratio (HR) 1.35, 95% CI 1.20–1.52) and TBI (HR 1.26, 95% CI 1.06–1.50) (Tables 2 and 3). The risk was highest at the beginning of antidepressant use (HR 1.71, 95% CI 1.10–2.66, for head injury, and HR 2.06, 95% CI 1.12–3.82, for TBI). For head injury, the risk remained elevated even until 2 years of use whereas, for TBI, the risk was significant only for the first 30 days of use, although the point estimate was indicative of an increased risk with longer durations of use. SSRI use was associated with an increased risk of head injury (HR 1.26, 95% CI 1.10–1.45), whereas drug class analyses for TBI showed no risk for either drug classes. Antidepressants were associated with both outcomes in as-treated and intention-to-treat analyses for the first 180 days of use, although the confidence intervals for TBI also included 1. No significant dose-response for risk of head injuries or TBIs was found in analyses of dose categories (Additional file 1: Table S1).

**Table 2:** Antidepressant use and associated risk of head injury among persons with Alzheimer’s disease

	Number of events	Person years	Age-adjusted event rate per 100 person-years (95% CI)	Unadjusted HR (95% CI)	Adjusted HR (95% CI)^a^	Propensity score adjusted HR (95% CI)
Users compared with nonusers						
Nonusers	981	47,423	2.43 (2.06–2.35)	Reference	Reference	Reference
Users	392	13,178	2.98 (2.49–3.06)	1.38 (1.23–1.55)	1.36 (1.21–1.53)	1.35 (1.20–1.52)
Antidepressant use classified according to duration of use						
1–30 days	37	877	3.97 (2.70–5.25)	2.15 (1.35–3.41)	2.12 (1.33–3.36)	1.71 (1.10–2.66)
31–180 days	102	3152	3.09 (2.49–3.70)	1.27 (1.00–1.61)	1.25 (1.00–1.59)	1.35 (1.06–1.71)
181–365 days	84	2669	3.07 (2.41–3.73)	1.35 (1.05–1.75)	1.33 (1.03–1.72)	1.35 (1.05–1.75)
366–731 days	90	3289	2.64 (2.10–3.18)	1.29 (1.01–1.64)	1.27 (1.00–1.62)	1.28 (1.01–1.63)
> 731 days	79	3213	2.29 (1.56–3.01)	1.50 (1.17–1.93)	1.48 (1.15–1.91)	1.35 (1.05–1.74)
Drug class specific analyses (*n* = 38 concomitant users excluded)						
Other antidepressant	110	4465	2.24 (1.78–2.71)	1.01 (0.83–1.23)	0.95 (0.77–1.16)	0.94 (0.77–1.15)
SSRI	246	7664	2.74 (2.40–3.99)	1.32 (1.15–1.52)	1.28 (1.11–1.48)	1.26 (1.10–1.45)
As-treated analyses restricted to the first 180 days						
Nonusers	248	9758	2.33 (2.03–2.63)	Reference	Reference	Reference
Users	139	4032	3.29 (2.74–3.84)	1.36 (1.11–1.68)	1.31 (1.06–1.62)	1.34 (1.08–1.66)
Intention-to-treat analyses restricted to the first 180 days						
Nonusers	268	10,226	2.35 (2.05–2.65)	Reference	Reference	Reference
Users	173	5060	3.25 (2.76–3.75)	1.31 (1.08–1.58)	1.26 (1.03–1.53)	1.34 (1.10–1.64)

The reference category in all analyses is nonuse

^a^Adjusted for cardiovascular disease, diabetes, asthma/COPD, epilepsy, substance abuse, cancer, schizophrenia, bipolar disorder or depression, baseline use of antipsychotics, opioids and benzodiazepines and related drugs, history of stroke and hip fracture, and socioeconomic position

*CI* confidence interval, *HR* hazard ratio, *SSRI* selective serotonin reuptake inhibitor

**Table 3:** Antidepressant use and associated risk of traumatic brain injury among persons with Alzheimer’s disease

	Number of events	Person years	Age-adjusted event rate per 100 person-years (95% CI)	Unadjusted HR (95% CI)	Adjusted HR (95% CI)^a^	Propensity score adjusted HR (95% CI)
Users compared with nonusers						
Nonusers	492	47,514	1.10 (1.00–1.20)	Reference	Reference	Reference
Users	185	13,184	1.33 (1.13–1.53)	1.30 (1.10–1.54)	1.25 (1.05–1.49)	1.26 (1.06–1.50)
Antidepressant use classified according to duration of use						
1–30 days	21	877	2.27 (1.29–3.25)	2.13 (1.16–3.93)	2.06 (1.12–3.80)	2.06 (1.12–3.82)
31–180 days	46	3,154	1.39 (0.98–1.80)	1.26 (0.88–1.78)	1.21 (0.85–1.73)	1.22 (0.85–1.73)
181–365 days	40	2,678	1.52 (1.04–2.00)	1.25 (0.87–1.81)	1.21 (0.84–1.75)	1.21 (0.84–1.75)
366–731 days	47	3,291	1.38 (0.98–1.77)	1.41 (1.01–1.96)	1.35 (0.97–1.90)	1.36 (0.97–1.90)
> 731 days	31	3,214	0.64 (0.26–1.02)	1.11 (0.75–1.64)	1.07 (0.72–1.58)	1.07 (0.73–1.59)
Drug class specific analyses (compared with nonuse, *n* = 38 concomitant users excluded)						
Other antidepressant	50	4,461	1.08 (0.72–1.43)	0.91 (0.68–1.22)	0.87 (0.65–1.17)	0.86 (0.64–1.15)
SSRI	114	7,671	1.31 (1.06–1.56)	1.22 (0.99–1.49)	1.21 (0.99–1.49)	1.17 (0.95–1.44)
As-treated analyses restricted to the first 180 days						
Nonusers	117	9,759	1.13 (0.92–1.34)	Reference	Reference	Reference
Users	67	4,035	1.59 (1.20–1.97)	1.38 (1.02–1.87)	1.23 (0.90–1.67)	1.26 (0.92–1.71)
Intention-to-treat analyses restricted to the first 180 days						
Nonusers	129	10,254	1.19 (0.98–1.40)	Reference	Reference	Reference
Users	89	5,084	1.66 (1.31–2.01)	1.39 (1.06–1.83)	1.28 (0.97–1.69)	1.31 (1.00–1.73)

The reference category in all analyses is nonuse

^a^Adjusted for cardiovascular disease, diabetes, asthma/COPD, epilepsy, substance abuse, cancer, schizophrenia, bipolar disorder or depression, baseline use of antipsychotics, opioids and benzodiazepines and related drugs, history of stroke and hip fracture, and socioeconomic position

*CI* confidence interval, *HR* hazard ratio, *SSRI* selective serotonin reuptake inhibitor

In case-crossover analyses, antidepressant use was consistently associated with higher risk of head injury in all control periods, adjusted odds ratios (ORs) ranging from 1.64 to 2.04 (control periods 30-45, 60–74 and 120–134 days, respectively; Table 4). For TBI, antidepressant use was significantly associated with an increased risk only with the control period 120–134 days (OR 1.81, 95% CI 1.22–2.69), although the point estimates were indicative of the increased risk.

**Table 4:** Sensitivity analyses for risk of head injuries (*n* = 3838) and brain injuries (*n* = 1914) associated with antidepressant use in case-crossover design

	Unadjusted OR (95% CI)	Adjusted OR (95% CI)^a^
Case window 1–14 days before the head injury		
Control window 30–45 days before	1.77 (1.13–2.77)	1.71 (1.09–2.68)
Control window 60–74 days before	1.67 (1.19–2.34)	1.64 (1.17–2.30)
Control window 120–134 days before	2.04 (1.55–2.70)	2.04 (1.55–2.70)
Case window 1–14 days before the traumatic brain injury		
Control window 30–45 days before	1.53 (0.83–2.82)	1.46 (0.79–2.70)
Control window 60–74 days before	1.36 (0.85–2.16)	1.33 (0.83–2.11)
Control window 120–134 days before	1.84 (1.24–2.73)	1.81 (1.22–2.69)

^a^Adjusted for time-dependent use of benzodiazepines and related drugs, antipsychotics, and opiods

*CI* confidence interval, *OR* odds ratio

## Discussion

To our knowledge, this is the first study to assess the risk of head and brain injuries associated with antidepressant use. Antidepressant users had an increased risk of head injuries and TBIs among persons with Alzheimer’s disease in the exposure-matched cohort design while adjusting for propensity score. The risk was highest at the beginning of antidepressant use and, for head injuries, lasted for over 2 years of use and, for traumatic brain injury, the risk was evident only at the beginning of use although the risk estimates were also suggestive of increased risk after that. As-treated and intention-to-treat analyses for the first 180 days resulted in similar results as for the main analyses. Sensitivity analyses with within-individual case-crossover design indicated that antidepressant use is associated with an increased risk of head injuries but not consistently with TBIs. In general, the associations between antidepressant use and TBIs were less consistent and some analyses lacked statistical significance although the risk estimates indicated an increased risk, possibly due to small number of events for users.

Our study population included persons with Alzheimer’s disease who are at increased risk of falling compared with cognitively intact older persons [21]. Previous studies also show that persons with AD have two- to three-times higher risk of injurious falls such as hip fractures [22, 23]. Previous studies have indicated that antidepressant use is associated with an increased risk of falls and fractures among older persons [8–10]. Our results on an increased risk of head and brain injuries which typically are caused by falls are in line with these previous results. As antidepressant use has been associated with an increased risk of falling among the general older population it is likely that the risk of head and brain injuries is not limited to persons with AD or dementia and further studies should be conducted among older persons without dementia/AD. Our findings are particularly concerning in the light of recent studies reporting an increasing trend of antidepressant use among persons with dementia [2, 7]. These trends imply that antidepressants are used as the “safer choice” instead of antipsychotics for various BPSD symptoms, and this treatment practice may prove problematic.

The mechanisms behind the high risk for injurious falls are assumed to be related to the fall-risk increasing features of antidepressants [8], as the majority of head and brain injuries are caused by falls among older persons [12]. Sedative drug use has been associated with slower walking speed, impaired balance [24], and lower strength among older persons [25]. These findings may partially be explained by the sedative effects of antidepressants to various receptor activities in the central nervous system. The second-generation antidepressants antagonize H1 and alpha2-receptors leading to sedation [26, 27]. Antagonism of muscarinic receptors is known to cause sedative effects [26], although tricyclics were infrequently used in our study. Antidepressant use is shown to impair cognitive processing and produce impairments in alertness, reaction time, and motor abilities, and these have been also associated with SSRIs [28]. In addition, antidepressants are also known to increase the risk of hyponatremia [9, 29] which may lead to falls [30].

For head injuries, SSRI use was associated with an increased risk whereas other antidepressants were not significantly associated. For TBI, neither SSRI or other antidepressants resulted in significant associations possibly due to the smaller number of TBIs. It is unlikely that the risk of head injuries would be limited to SSRIs. Many antidepressants in the “other antidepressant” category, such as mianserine, mirtazapine, and some tricyclic antidepressants, have more pronounced sedative effects than SSRIs [26, 28]. In the study by Coupland et al., SSRIs were associated with a somewhat higher risk of falls than other antidepressants but a lower risk of many other adverse outcomes, and a similar risk of fractures [9]. Thus, differential risks associated with antidepressant classes should be further studied in future.

## Strengths and limitations

We investigated antidepressant use and the associated risk of head and brain injuries in a large, nationwide cohort including community-dwelling persons with Alzheimer’s disease. The results are generalizable to community-dwelling persons with AD. The analyses were restricted to the first head or brain injury to avoid multiple hospitalizations due to the same event. The new user design, with exclusion of prevalent users, controls for survival and selection biases associated with prevalent use. As aging and the progression of AD increases the risk of head and brain injuries, we formed an exposure-matched cohort design by matching nonusers to every antidepressant initiator. With this design, we were able to control for time since AD diagnosis which is a proxy for progression of the disease. We also matched comparison persons in terms of age since age is a major risk factor for falling [31]. Besides having age, gender, and time since AD diagnosis utilized in the matching, the analyses were adjusted for propensity score predicting antidepressant treatment and propensity score adjusted analyses confirmed the increased risk. Intention-to-treat analyses controlled for informative censoring and led to similar results. As in all observational studies, residual confounding may still exist. It is possible that the indications of antidepressant use may partially explain the observed association. In the observational study setting we lacked precise knowledge on indications for drug use, and the severity, frequency, and duration of symptoms for which antidepressants were used. However, sensitivity analyses with a case-crossover design were conducted to further assess unmeasured confounding at an individual level, such as problems with balance or mobility, and capabilities for activities of daily functioning. The sensitivity analyses confirmed results for head injuries and partly for TBIs.

We utilized a mathematical modeling method, PRE2DUP [19], to construct drug-use periods from Prescription register data to retrieve valid estimates of drug exposure [32]. Previous research has demonstrated that the Prescription register provides valid data for antidepressant use among older persons [33]. Due to limitations in the registers (i.e., lack of data from outpatient care), head and brain injuries are limited to the cases treated in hospitals or who died due to injury. Thus, milder events may be lacking and the risks may be underestimated. Although registers widely cover data on important confounders, many important factors, such as the severity of AD, frequency and nature of behavioral symptoms, or indication for drug use, are not recorded and, thus, residual confounding may exist. These factors were controlled for to some extent in within-individual analyses.

## Conclusions

Antidepressant use has been previously associated with an increased risk of falls, but our novel findings indicate that they are associated with severe injurious falls, i.e., those resulting in head or brain injuries among persons with Alzheimer’s disease. The association between antidepressant use and head and brain injuries should be confirmed in further studies. As antidepressant use has also been associated with an increased risk of falling in previous studies, clinicians should keep on carefully considering indications and use of antidepressants for the safety of vulnerable patients.

## Additional file

Additional file 1: Detailed description of covariates. Covariates in the propensity score. **Table S1**. Risk of head and brain injury associated with antidepressant monotherapy according to dose categories. (DOC 42 kb)
