# Supplementary material for: Risk of head and traumatic brain injuries associated with antidepressant use among community-dwelling persons with Alzheimer’s disease: a nationwide matched cohort study
Source: Alzheimers Res Ther. 2017 Aug 1;9:59. doi: 10.1186/s13195-017-0285-3 (PMC5540546; doi:10.1186/s13195-017-0285-3)
Supplement: Additional file 1: — Detailed description of covariates. Covariates in the propensity score. Table S1. Risk of head and brain injury associated with antidepressant monotherapy according to dose categories. (DOC 42 kb) [file 13195_2017_285_MOESM1_ESM.doc]

**Additional file 1**

**Detailed description of covariates**

Data on drug use were obtained from the Prescription register and included antipsychotics (ATC class N05A excluding lithium), opioids (N02A) and benzodiazepines and related drugs (BZDRs, N05BA, N05CD, N05CF), bisphosphonates (M05BA, M05BB), anti-parkinson drugs (N04), antiepileptics (N03), analgesics (non-steroidal anti-inflammatory drugs M01A excluding glucosamine, and paracetamol N02BE01) and cardiovascular drugs (C*). Use of these drugs were modelled in the same way as antidepressant use.

Covariates from Special Reimbursement register data (since 1972 until the start of the follow-up) included chronic diseases that are assigned with special reimbursement code to get higher reimbursement of drugs. Diagnostic protocol for these diseases is pre-defined and monitored by the Social Insurance Institution of Finland similarly as for diagnoses of Alzheimer’s disease. Diagnoses in the Special Reimbursement register represent diagnoses made both in in-and outpatient care, and included cardiovascular diseases, diabetes, epilepsy, and asthma/ chronic obstructive pulmonary disease (COPD). Cardiovascular diseases were defined as having one or several of the following: chronic heart failure, arterial hypertension, coronary artery disease and chronic arrhythmia.

The following diagnoses were derived from Hospital discharge register (data since 1972 until the start of the follow-up) and thus, are based on inpatient diagnoses only: substance abuse, stroke, hip fracture, schizophrenia and bipolar disorder/ depression. These comorbidities were based on ICD-10 codes with corresponding ICD-8 and -9 codes. History of substance abuse was defined as hospitalization based on the diagnosis of mental and behavioral disorders due to psychoactive substance (ICD F10-F19) or alcoholic pancreatitis (K85.2) or substance abuse indicated as the reason for hospital admission. History of stroke (I60-64) and hip fracture (S72.0-72.2) were retrieved similarly. History of psychiatric disorders (schizophrenia, schizotypal or delusional disorders F20-29, and bipolar disorder/ depression F30-34, F38-39) were restricted to diagnoses since 1972 until 5 years before the diagnosis of Alzheimer’s disease. The 5-year gap before the diagnosis was inserted to exclude treatment periods for prodromal symptoms of Alzheimer’s disease.

Socioeconomic position was defined as the highest occupational position recorded for study participants in their middle age (45-55 years old), according to classification by Statistics Finland and categorized to four classes (high, medium, low, unknown). The highest class included entrepreneurs and higher clerical workers, medium class included lower clerical workers and employees, and the lowest class included unemployed, retired and students. Persons with unknown socioeconomic class and those with missing data at Statistics Finland (about 1% of the cohort) were combined to class “unknown”. Other variables did not include missing data.

Covariates selected for traditional adjustments were cardiovascular disease, diabetes, asthma/COPD, epilepsy, substance abuse, cancer, schizophrenia, bipolar disorder or depression, baseline use of antipsychotics, opioids and benzodiazepines and related drugs; history of stroke and hip fracture, and socioeconomic position. Drug use was measured at the start of the follow-up (drug used at the index date) whereas comorbidities were measured since the beginning of the register 1972 until the start of the follow-up (except for psychiatric disorders, with a 5-year gap before the index date).

**Covariates in the propensity score**

Propensity score for antidepressant treatment was derived by logistic regression. Covariates associated with antidepressant use in univariate analyses and predictors of antidepressant use and falling according to previous studies were considered for propensity score. In addition to covariates included in the traditional adjustments, drug use was also assessed as “ever” use of certain drugs since 1995. Ever use of opioids, antipsychotics, BZDRs, antidepressants, bisphosphonates, anti-parkinson drugs, antiepileptics, analgesics and cardiovascular drugs were included, in addition to baseline use of opioids, antipsychotics and BZDRs. Additional comorbidities from Hospital Discharge register (recorded ever before the start of the follow-up) included in the propensity score were metastatic cancer, chronic heart failure, renal failure, hemiplegia, alcohol abuse, any tumor, cardiac arrhythmia, any chronic pulmonary disease, coagulopathy, complicated diabetes, anaemia, fluid and electrolyte disorders, liver disease, peripheral vascular disorder, psychosis, pulmonary circulation disorders, hypertension, and previous hospital-treated fractures.

| **Table S1.** Risk of head and brain injury associated with antidepressant monotherapy according to dose categories. | | | |
| --- | --- | --- | --- |
|  | **Number of events** | **Person-years** | **Unadjusted HR (95% CI)** |
| **Head injury** | | | |
| Dose categories compared with no use | | | |
| Nonuse | 969 | 40191 | ref |
| <1 DDDs | 312 | 10827 | 1.22 (1.04-1.43) |
| ≥1 DDDs | 44 | 1302 | 1.40 (0.91-2.16) |
| Higher dose compared with lower dose among users | | | |
| <1 DDDs | 312 | 10827 | ref |
| ≥1 DDDs | 44 | 1302 | 1.18 (0.86-1.62) |
| **Traumatic brain injury** | | | |
| Dose categories compared with no use | | | |
| Nonuse | 484 | 40094 | ref |
| <1 DDDs | 145 | 10826 | 1.14 (0.91-1.42) |
| ≥1 DDDs | 19 | 1306 | 1.37 (0.73-2.54) |
| Higher dose compared with lower dose among users | | | |
| <1 DDDs | 145 | 10826 | ref |
| ≥1 DDDs | 19 | 1306 | 1.10 (0.68-1.77) |
